# Quantitative Analysis of Biomarkers to Distinguish Between Korean and Chinese Mud Loaches

**DOI:** 10.3390/foods15020304

**Published:** 2026-01-14

**Authors:** Hyunsuk Kim, Junho Yang, Hyunji Lee, Hyeyoung Lee, Jiyoung Shin, Ji-Young Yang

**Affiliations:** 1 Advanced Analysis Division, Toxicological Evaluation and Research Department, National Institute of Food Drug Safety Evaluation, Ministry of Food and Drug Safety, Cheongju-si 28159, Republic of Korea; zrepsriy@naver.com; 2 Department of Food Science & Technology, Pukyong National University, Busan 48513, Republic of Korea; yjunho9@gmail.com; 3 Department of Applied Chemistry-Food Science and Technology, Dong-Eui University, Busan 47340, Republic of Korea; guswl4354@naver.com (H.L.); hlee@deu.ac.kr (H.L.)

**Keywords:** *Misgurnus mizolepis*, origin discrimination, targeted metabolomics, biomarker assessment, optimal cut off

## Abstract

Mud loach (*Misgurnus mizolepis*) is a freshwater fish widely farmed in inland aquaculture owing to its nutritional value. However, failure to distinguish Chinese from Korean mud loach negatively affects the distribution economy and food safety regulation. Untargeted profiling was previously used to determine the origin of mud loaches, and N-acetylhistidine and anserine were selected as biomarker candidates. However, their quantitative verification and practical applicability for origin discrimination have not been thoroughly investigated. In this study, mud loaches of different geographical origins were analyzed using liquid chromatography-ultraviolet and liquid chromatography-tandem mass spectrometry to quantify the two metabolites, followed by statistical and receiver operating characteristic (ROC) analyses to evaluate their discriminative performance. Compared with Korean mud loaches, Chinese mud loaches showed significantly higher concentrations of both metabolites. The area under the curve values for N-acetylhistidine and anserine were 0.88 and 0.89, respectively, reflecting high sensitivity and specificity for discriminating between Korean and Chinese mud loaches. Cutoff values were established for reliably distinguishing the geographical origin of mud loaches. The established approach based on N-acetylhistidine and anserine can be used to determine the geographical origin of mud loach.

## 1. Introduction

The mud loach (*Misgurnus mizolepis*) of the family Cobitidae predominantly inhabits muddy substrates of rivers and agricultural waterways throughout South Korea [1,2]. It is widely consumed because of its substantial nutritional content, including proteins and vitamins, and distinctive flavor [3,4,5]. However, the mud loaches consumed in South Korea are predominantly imported from China, comprising nearly 80% of the total market volume [6,7]. Moreover, imported and domestic mud loaches are difficult to distinguish because of their morphological similarities, posing risks to economic fairness and food safety. Additionally, ofloxacin, which is not permitted for use in aquaculture species in South Korea, has been detected in certain imported mud loaches. Recently, the geographical origin authentication of food has gained increasing importance in terms of preventing food fraud, ensuring quality control, and enhancing consumer trust [8,9]. Thus, accurate verification of the geographical origin of Korean and Chinese mud loaches is crucial.

Metabolomics has been increasingly applied to determine the geographic origins of different products in the food industry, including agricultural [10], aquatic [11,12], and livestock products [13]. Metabolomics is a robust alternative to traditional documentation-based tracking and DNA-based identification [14]. Untargeted metabolomics is widely applied for comprehensive metabolic profiling and the discovery of potential biomarkers by screening a broad range of metabolites [15,16]. However, because this approach primarily focuses on pattern recognition, subsequent validation steps are required to address challenges such as metabolite misidentification, matrix effects, and limited reproducibility in practical applications [17,18]. In this context, targeted metabolomics plays a complementary role by enabling the accurate and reproducible quantification of selected metabolites and the elucidation of specific metabolic pathways. In particular, triple quadrupole-based selected reaction monitoring (SRM) using liquid chromatography–tandem mass spectrometry (LC-MS/MS) provides excellent sensitivity and precision for the quantitative validation of predefined biomarkers [19]. Although targeted analyses are inherently limited in scope and may overlook unexpected metabolites, they are well suited for confirmatory measurements once candidate biomarkers have been identified. Consequently, metabolomic authentication strategies benefit from a sequential two-stage workflow comprising untargeted biomarker discovery followed by targeted quantitative verification. Nevertheless, studies that systematically integrate these two complementary metabolomic approaches for origin authentication remain scarce [17].

A previous study used untargeted metabolomics to investigate the metabolic differences between Korean and Chinese mud loaches [20]. Among the identified metabolites, N-acetylhistidine and anserine emerged as biomarker candidates for differentiating Korean and Chinese mud loaches. These results were based on multivariate statistical analyses, including principal component analysis (PCA) and orthogonal partial least squares-discriminant analyses (OPLS-DA), which demonstrated clustering between the two groups. Furthermore, the area under the curve (AUC) values obtained from receiver operating characteristic (ROC) curve analysis substantiated the classification performance of these biomarkers.

Therefore, this study combined untargeted and targeted metabolomics by applying N-acetylhistidine and anserine using high-performance liquid chromatography-ultraviolet (HPLC-UV) and LC-MS/MS in Korean and Chinese mud loaches. We hypothesized that establishing validated methods for these metabolites would effectively distinguish the geographical origin of mud loach.

## 2. Materials and Methods

### 2.1. Chemicals and Reagents

HPLC-grade formic acid, methanol, acetonitrile, isopropyl alcohol, and water were purchased from Honeywell Burdick and Jackson (Muskegon, MI, USA). Ammonium acetate, trichloroacetic acid (TCA), N-acetylhistidine, and (R)-phenylephrine hydrochloride (PEH; internal standard) were obtained from Sigma-Aldrich (Saint Louis, MO, USA). Anserine was purchased from MedChemExpress (Monmouth Junction, NJ, USA).

### 2.2. Sample Collection and Preparation

Mud loach samples from Korea and China were collected from commercial aquaculture farms, respectively. Overall, 40 samples were obtained per season, consisting of five individuals from each origin (Korean and Chinese), ensuring balanced seasonal representation. Korean samples were cultivated from inland aquaculture facilities in Yangpyeong (Gyeonggi-do) and Namwon (Jeollanam-do), whereas Chinese samples originated from aquaculture farms in Jiangsu and Jiangxi provinces. Prior to analysis, the total length and body weight of each mud loach were measured. The Korean mud loaches had an average total length of 15.8 ± 2.5 cm and average weight of 21.5 ± 8.3 g, whereas the Chinese mud loaches had an average total length of 16.0 ± 1.7 cm and average weight of 25.0 ± 8.1 g. Muscle tissue was subsequently collected from the fish samples for metabolite extraction. The animal study protocol was reviewed and approved by the Pukyong National University-Institutional Animal Care and Use Committee on ethical procedures and scientific care (approval number: PKNUIACUC-2022-30). The collected muscle tissue was freeze-dried for 48 h, vacuum-packed, and stored at −80 °C until further analysis.

### 2.3. Determination of N-Acetylhistidine Using LC-UV

N-acetylhistidine was identified using a reversed-phase HPLC system equipped with a UV detector. The analytical procedure was based on previously reported methods with slight modifications [21,22]. The sample (0.5 g) was homogenized using 10 volumes of 80% ethanol and then centrifuged at 2000× *g* for 20 min. The supernatants were dried using a speed vacuum concentrator, reconstituted in phosphate buffer (pH 2.0), and then filtered through a 0.45 μm PTFE filter (Advantec, Tokyo, Japan). Chromatographic analysis was conducted using a Dionex Ultimate 3000 UHPLC system (Thermo Fisher Scientific, Waltham, MA, USA) equipped with an Acclaim C18 column (4.6 mm × 250 mm, 5 μm; Thermo Fisher Scientific). Isocratic elution was performed using 0.1 M phosphate buffer (pH 2.0) as the mobile phase at a flow rate of 0.6 mL/min. The injection volume was 10 μL, and detection was conducted at a wavelength of 210 nm. N-acetylhistidine was identified and quantified based on the retention time and UV absorbance relative to a reference standard.

### 2.4. Determination of Anserine Using LC-MS/MS

Anserine was detected using LC-MS/MS following the method described by Lee et al. [23], which was previously validated for the quantitative analysis of anserine in seafood matrices. The sample (0.5 g) was homogenized and filtered through a 60-mesh (250 μm) sieve (Chung Gye Sang Gong Sa, Seoul, Republic of Korea). Subsequently, 50 μL of internal standard PEH (0.25 mg/mL) was added to each homogenate. Anserine was extracted with 7 mL of 10% TCA, sonicated for 5 min, and incubated at 25 °C for 20 min. The mixtures were centrifuged at 10,000× *g* for 15 min at 4 °C, and the supernatant was collected. This extraction step was repeated once, and the combined supernatants were adjusted to a total volume of 25 mL with the extraction solvent. The extract was diluted 20-fold with 75% acetonitrile and filtered through a 0.2 μm PTFE filter (Whatman, Maidstone, UK). The filtrates were transferred to injection vials for LC-MS/MS analysis using a Thermo Fisher TSQ Endura triple-stage quadrupole mass spectrometer coupled with an Ultimate 3000 UHPLC system (Thermo Fisher Scientific, Waltham, MA, USA). Chromatographic separation was performed using an Acquity UPLC BEH HILIC analytical column (Waters; 2.1 mm × 50 mm, 1.7 μm) maintained at 40 °C. The mobile phase consisted of 10 mM ammonium acetate in acetonitrile/water (50:50, *v*/*v*) as eluent A and 10 mM ammonium acetate in acetonitrile/water (75:25, *v*/*v*) as eluent B. The gradient elution program was as follows: 0–10 min, 100% B; 10–10.1 min, 0% B; 10.1–15 min, 0% B; 15–15.1 min, 100% B; and 15.1–25 min, 100% B. The flow rate was 0.2 mL/min, and the injection volume was 2 μL. Mass spectrometric detection was conducted using an electrospray ionization source in positive ion mode under the following conditions: spray voltage, 3500 V; ion transfer tube temperature, 350 °C; and vaporizer temperature, 275 °C. The analysis was conducted in the SRM mode. SRM transitions, including the collision energy and RF lens settings, are summarized in Table 1. Data acquisition and processing were performed using Thermo Xcalibur Quan Browser software version 2.0 (Thermo Fisher Scientific, Waltham, MA, USA).

### 2.5. Data Processing and Statistical Analysis

Univariate statistical analyses were performed using Student’s *t*-test to evaluate differences in metabolite concentrations between the Korean and Chinese mud loach samples. *p*-values were adjusted for multiple comparisons using the Benjamini–Hochberg false discovery rate (FDR) method. ROC curve analysis, a widely used method for visualizing and quantifying the performance of decision models, was conducted using MetaboAnalyst v6.0 (www.metaboanalyst.ca) to assess the discriminatory ability of the selected metabolites and to evaluate whether their classification performance was maintained across different seasons [24,25]. The area under the curve (AUC) values and 95% confidence intervals (CIs) were calculated via bootstrap resampling. Optimal cutoff values were determined by maximizing the sensitivity and specificity based on Youden’s index.

## 3. Results

### 3.1. Identification of Biomarker Compounds in Mud Loach

**Figure 1:**
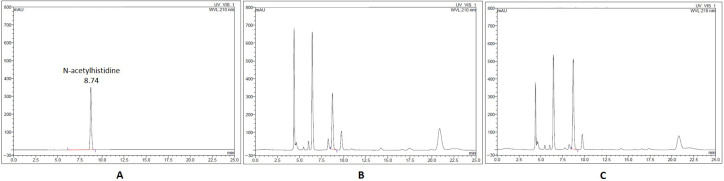
LC-UV chromatograms of N-acetylhistidine (**A**) standard, (**B**) Korean mud loach, (**C**) Chinese mud loach.

**Figure 2:**
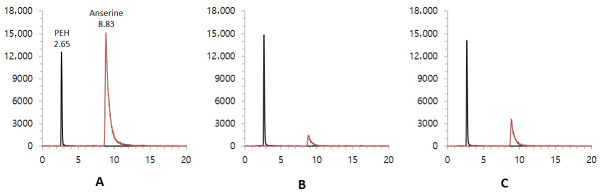
LC-MS/MS chromatograms of anserine (**A**) standard, (**B**) Korean mud loach, (**C**) Chinese mud loach.

The presence of N-acetylhistidine and anserine in Korean and Chinese mud loaches was investigated to assess the suitability of these compounds as biomarkers for origin classification. All samples showed clear chromatographic peaks without interference from matrices. N-acetylhistidine was analyzed using LC-UV detection. The reference standard exhibited a retention time of approximately 8.7 min. Corresponding peaks with comparable shapes appeared reproducibly in the Korean and Chinese samples at identical retention times (Figure 1). Anserine was analyzed using LC-MS/MS in positive ionization SRM mode. The standard compound was detected at a retention time of approximately 8.83 min with a transition of *m*/*z* 241 → 109.1. The internal standard PEH was also detected at *m*/*z* 168.1 → 150.1 with a retention time of 2.65 min (Figure 2). The same peaks were consistently observed in the samples under identical conditions. Both compounds exhibited sharp and distinct chromatographic peaks, confirming the robustness of the instrumental conditions. Although these compounds were originally noted in untargeted metabolomic studies, targeted assessment in this study enabled unambiguous detection, strengthening the credibility of the analysis. These results can be used as a foundation for origin-based statistical evaluations.

### 3.2. Comparative Quantification Between Korean and Chinese Samples

Quantitative analyses were conducted to determine the concentrations of N-acetylhistidine and anserine in Korean and Chinese samples. Statistical differences in metabolite concentrations between Korean and Chinese mud loaches were evaluated to confirm their effectiveness as geographical biomarkers (Table 2). The mean concentration of N-acetylhistidine was 31.9 ± 18.8 mg/100 g in the Korean mud loaches and 64.6 ± 17.3 mg/100 g in the Chinese mud loaches. Statistical analysis using Student’s *t*-test revealed a significant difference between the Korean and Chinese groups (*p* = 2.1 × 10^−6^), with the Chinese samples exhibiting approximately 2.02-fold higher concentrations than that of the Korean samples. The mean concentration of anserine was 5.5 ± 2.0 mg/100 g in the Korean mud loaches and 10.0 ± 3.0 mg/100 g in the Chinese mud loaches, with a significant difference between groups (*p* = 2.2 × 10^−6^). The Chinese samples exhibited approximately 1.84-fold higher concentrations than the Korean samples.

### 3.3. Evaluation of Discrimination Models for Origin Classification

**Figure 3:**
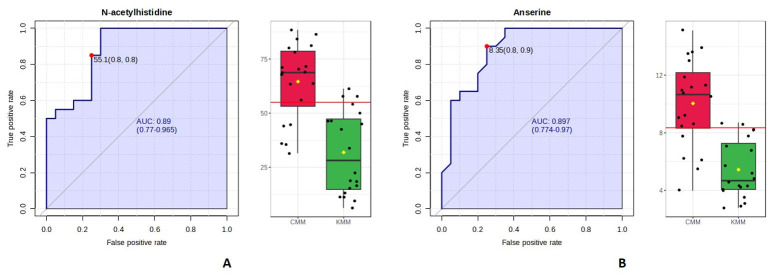
Receiver operating characteristic (ROC) curve of N-acetylhistidine (**A**) and anserine (**B**) in Korean (KMM) and Chinese mud loach (CMM).

**Figure 4:**
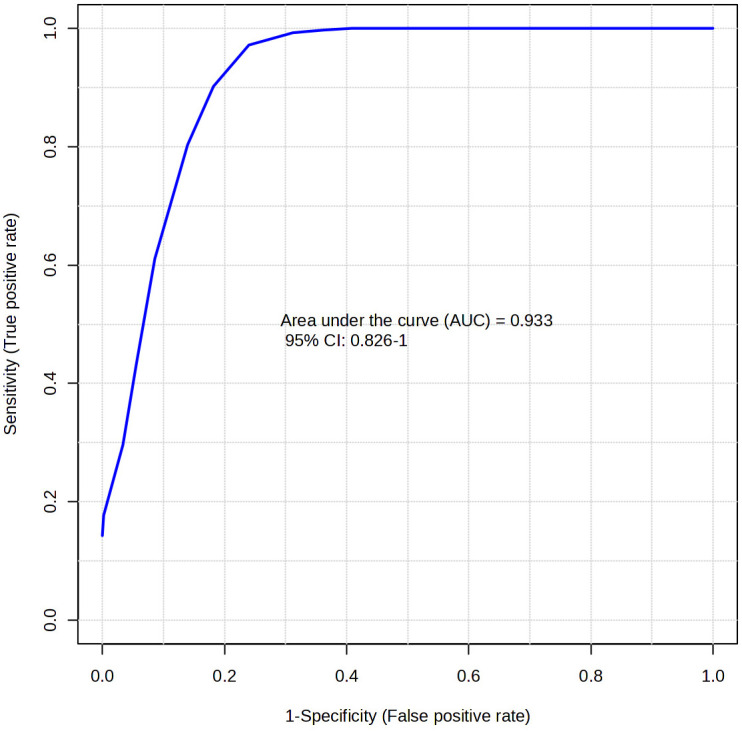
Multivariate ROC curve of N-acetylhistidine and anserine to discriminate between Korean (KMM) and Chinese mud loach (CMM).

In this study, N-acetylhistidine and anserine were assessed using the classical univariate ROC module to determine their individual effectiveness in geographical origin discrimination, without the influence of multivariate modeling. The AUC value, calculated as the area under the ROC curve, ranges from 0 to 1; the higher the value, the better the model performs. According to established guidelines, AUC values are interpreted as follows: 0.9–1.0 = excellent, 0.8–0.9 = good, 0.7–0.8 = fair, 0.6–0.7 = poor, and 0.5–0.6 = fail. AUC values > 0.5 are generally regarded as demonstrating meaningful diagnostic capacity [26]. The AUC values for N-acetylhistidine and anserine were 0.88 and 0.89, respectively, both of which meet the “good” range in accordance with established guidelines (Figure 3). To ensure statistical robustness, 95% CIs were estimated using bootstrap resampling with 500 iterations. The resulting 95% CIs were 0.770–0.965 for N-acetylhistidine and 0.774–0.970 for anserine, supporting their ability to distinguish between Korean and Chinese samples [27,28]. The multivariate ROC curve constructed using the two selected metabolites yielded an AUC of 0.93 with a 95% confidence interval of 0.826–1.000 (Figure 4).

Additionally, ROC analysis provides a critical threshold, which is derived from the balance between sensitivity and specificity. The cutoff value represents the concentration boundary used to distinguish samples based on their geographical origin and directly impacts the practical usability of the decision model. Youden’s index is commonly employed to determine the optimal cutoff by maximizing the combined sensitivity and specificity [29]. Sensitivity refers to the proportion of correctly identified positive cases (true positives), whereas specificity refers to the proportion of correctly excluded negative cases (true negatives). High values for both parameters suggest enhanced separation power in origin determination [30].

Based on Youden’s index, the optimal cutoff value for N-acetylhistidine was 55.1 mg/100 g, achieving sensitivity and specificity values of 0.85 and 0.75, respectively, thereby correctly classifying 85% and 75% of the Chinese and Korean samples, respectively. The optimal cutoff for anserine was 8.4 mg/100 g, with a sensitivity of 0.90 and specificity of 0.75, suggesting that anserine offers high sensitivity for identifying Chinese mud loaches.

### 3.4. Verification of Seasonal Stability in Biomarkers

The composition of fish changes according to the period and climatic environments, such as spawning season and water temperature [31]. Seasonal comparisons were performed to evaluate whether origin-dependent differences were maintained throughout the year (Table 3). Both metabolites consistently showed higher concentrations in the Chinese samples across all four seasons. N-acetylhistidine showed a fold change of 1.40–5.65 (Chinese/Korean) across the four seasons, accompanied by consistently significant *p*-values ranging from 8.6 × 10^−6^ to 8.8 × 10^−3^. The strongest separation was observed in spring and summer. The FDR-adjusted *p*-values (FDR = 3.4 × 10^−5^ to 1.2 × 10^−2^), calculated using the Benjamini–Hochberg procedure, remained below the 0.05 threshold in all seasons, indicating strong statistical significance and stable discrimination throughout the year [32]. Anserine showed a fold change of 1.14–3.64 across all seasons, with significant differences supported by *p*-values ranging from 4.0 × 10^−7^ to 4.7 × 10^−2^. The largest divergence occurred in summer and autumn. The corresponding FDR-adjusted values (FDR = 3.0 × 10^−7^ to 4.7 × 10^−2^) also remained below 0.05, confirming that anserine preserved meaningful origin-dependent separation regardless of seasonal variation.

**Figure 5:**
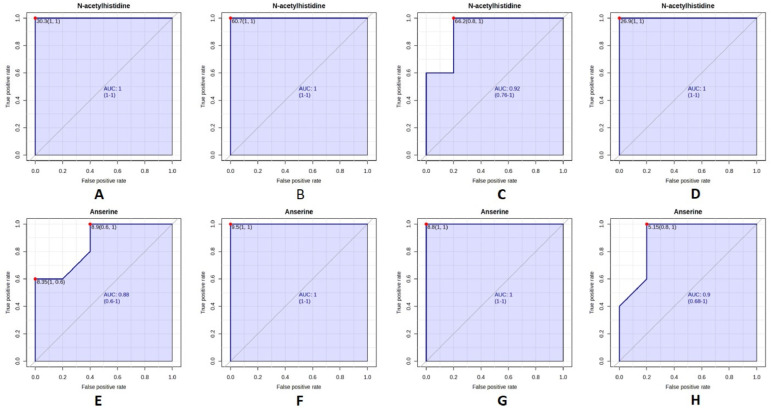
ROC curves of N-acetylhistidine and anserine for differentiating Korean and Chinese mud loach samples across seasons. Panels (**A**–**D**) represent N-acetylhistidine ((**A**), spring; (**B**), summer; (**C**), autumn; (**D**), winter), and panels (**E**–**H**) represent anserine ((**E**), spring; (**F**), summer; (**G**), autumn; (**H**), winter).

Seasonal ROC analyses were performed for each metabolite (Figure 5). N-acetylhistidine consistently exhibited AUC values above 0.9 in all seasons, indicating excellent discriminative ability and minimal seasonal sensitivity. Anserine also maintained high performance, with AUC values exceeding 0.85 across all seasons. No substantial seasonal decline in classification accuracy was observed for either biomarker.

## 4. Discussion

Distinguishing the geographical origin of mud loach is an important challenge in aquaculture, as the inability to differentiate between Korean and Chinese fish negatively affects distribution efficiency and food safety regulation. Previous metabolomic studies proposed N-acetylhistidine and anserine as candidate biomarkers for origin discrimination because both metabolites participate in physiological processes that are influenced by environmental and aquaculture conditions [20]. N-acetylhistidine, which is abundant in fish muscle, acts as an osmolyte and is involved in histidine-related energy metabolism; its levels fluctuate with nutrient availability and water temperature. Anserine, a major histidine-containing dipeptide, contributes to intracellular pH buffering, antioxidant defense, and muscle contractile regulation. Given these biochemical functions, both metabolites are likely to reflect differences in feeding practices, thermal environments, and production systems between Korea and China, thereby providing a mechanistic basis for their discriminatory potential. Nevertheless, despite their repeated identification in untargeted metabolomic studies, quantitative confirmation of these metabolites has remained limited.

In this study, N-acetylhistidine and anserine were quantitatively validated as biomarkers for distinguishing the geographical origin of mud loaches. LC-UV and LC-MS/MS analyses consistently detected both metabolites, revealing 2.02- and 1.84-fold significantly higher mean concentrations of N-acetylhistidine and anserine (*p* < 0.001), respectively, in the Chinese samples than in the Korean samples. Nevertheless, relatively large variations in N-acetylhistidine content were observed among the Chinese samples, which may reflect industrial impacts arising from diverse aquaculture systems and environmental conditions [33,34]. The ROC curve analysis showed AUC values of 0.88 and 0.89 for N-acetylhistidine and anserine, respectively, reflecting strong classification performance. In addition, combining the two metabolites in a multivariate model increased the AUC to 0.93, suggesting that their joint application can further strengthen origin-discriminatory performance.

These results should be interpreted considering extrinsic environmental and physiological factors. Previous investigations have shown that N-acetylhistidine concentrations increase by 24% within 2 h following the transfer of fish from freshwater to seawater, suggesting that external osmotic pressure rapidly affects N-acetylhistidine accumulation [35]. Moreover, N-acetylhistidine concentrations are significantly higher in fish inhabiting colder environments, reinforcing its role as an ambient indicator of water temperature and ecological variation [36]. In contrast, nutritional deprivation has been reported to induce the mobilization of muscular imidazole compounds, including histidine-related metabolites, leading to their reduction during starvation conditions [37]. These results indicate that N-acetylhistidine is highly responsive to extrinsic conditions, potentially contributing to regional, seasonal, and habitat-specific variations. Therefore, the largest origin-dependent differences were observed in spring and summer, when water temperature changes are substantial and periods of active growth, increased feeding, and pre-spawning physiological transitions coincide. Similarly, anserine is influenced by various environmental variables. Abe et al. [38] reported that anserine concentrations in tuna vary with starvation and exhaustion. Shima et al. [39] also reported that anserine concentrations are relatively higher in salmoniformes reared in low-flow lake environments, suggesting that differences in water quality and gut microbiota composition could affect anserine accumulation. Furthermore, Shima et al. [40] confirmed that increased water temperature directly elevates anserine concentrations and indirectly affects them through changes in metabolic cascades. Collectively, anserine is influenced by multiple factors, such as water temperature, farming environment, and microbiota composition. Accordingly, the origin-dependent differences in anserine were most pronounced in summer and autumn, when elevated water temperatures and increased growth or activity levels are expected to accentuate metabolic divergence. In this context, aquaculture systems in China are generally characterized by relatively warmer rearing conditions, prolonged transportation-associated starvation, and differences in feeding practices, whereas salinity conditions are broadly comparable to those in Korean freshwater aquaculture. Collectively, these contrasting aquaculture environments between Korea and China are likely to influence metabolic process in mud loach and contribute to the metabolomic differences observed in the present study.

The higher concentrations of N-acetylhistidine and anserine in Chinese samples than in Korean samples aligned with previously reported trends in untargeted metabolomic profiles [20]. Untargeted metabolomics provides a comprehensive approach to explore metabolite profiles and identify novel biomarker candidates. Targeted metabolomics enables consistent measurement procedures and the establishment of objective concentrations. A previous study employing ROC analysis based on quantitative data demonstrated that these biomarkers yield high AUC values and effectively differentiate between Korean and Chinese fish species [23]. The concordance between the non-targeted and targeted analyses shows that the differences in N-acetylhistidine and anserine concentrations are consistently observed across different analytical methods. This reproducibility suggests that the metabolic patterns reflect biological regulation according to the aquaculture environment of the origin. Therefore, linking untargeted and targeted metabolomics should be considered an essential step in advancing biomarker discovery.

Nevertheless, this study has limitations. The sample size was insufficient; thus, the generalization of the proposed cutoff values may be constrained. Future studies should conduct large-scale sample analyses that incorporate specimens from multiple distribution stages. Such studies could also verify the applicability of the proposed biomarkers in real-world import and distribution processes, thereby further advancing research on origin discrimination.

## 5. Conclusions

Our results demonstrate that the proposed biomarkers can be effectively applied in quantitative and statistical approaches for the geographical origin identification of mud loach. Both N-acetylhistidine and anserine clearly separated Korean and Chinese samples, confirming their suitability as origin-discriminating biomarkers based on reproducible quantitative measurements. The innovative aspect of this study resides in the targeted validation of N-acetylhistidine and anserine, which have been consistently identified in previous untargeted metabolomic studies, and in the establishment of statistically supported cutoff criteria for origin discrimination. By integrating quantitative analysis with ROC-based evaluation, this study extends biomarker discovery toward a more objective approach to origin verification. Accordingly, the application of defined cutoff values can reduce misclassification during distribution and ensure reliable country-of-origin labeling. Furthermore, this approach enables rapid verification and quality assurance, thereby improving distribution efficiency and strengthening regulatory oversight. As the first study to establish N-acetylhistidine and anserine as biomarkers for the geographical origin discrimination of mud loach through targeted analysis, the present work provides meaningful contributions from both academic and industrial perspectives and offers a scientific foundation for the development of verification methodologies and food traceability systems.

## Figures and Tables

**Table 1:** MS/MS parameters for the analysis of anserine and internal standard (IS) using LC–MS/MS.

Compound	Polarity	Precursor Ion (*m*/*z*)	Product Ion (*m*/*z*)	Collision Energy (V)	Log P	pKa	RF Lens
Anserine	+	241.1	109.1	23	−5.06	2.51, 6.97, 9.51	77
			170.1	16			
			168.1	13			
Phenylephrine HCl (IS)	+	168.1	150.1	10	−0.08	9.07, 9.67	35
			91.1	21			

**Table 2:** Comparison of N-acetylhistidine and anserine contents between Korean and Chinese mud loach.

Compound	Concentration, mg/100 g		Comparative Analysis	
	KMM	CMM	Ratio ^1^	*p*-Value ^2^
N-Acetylhistidine	31.9 ± 18.8	64.6 ± 17.3	2.02	2.11 × 10^−6^
Anserine	5.5 ± 2.0	10.0 ± 3.0	1.84	2.2 × 10^−6^

^1^ The ratio was calculated using the mean value. The values of the sample were CMM/KMM. ^2^ The *p*-value was obtained using Student’s *t*-test. KMM, Korean mud loach; CMM, Chinese mud loach.

**Table 3:** Seasonal comparison of N-acetylhistidine and anserine contents between Korean and Chinese mud loach.

Compound	Season	Concentration, mg/100 g		Comparative Analysis	
		KMM	CMM	*p*-Value	FDR
N-Acetylhistidine	Spring	11.3 ± 3.5	64.0 ± 10.0	8.6 × 10^−6^	3.4 × 10^−5^
	Summer	45.1 ± 7.7	76.6 ± 8.0	4.6 × 10^−4^	9.2 × 10^−4^
	Autumn	53.9 ± 5.3	75.6 ± 11.4	0.0088	0.0117
	Winter	17.2 ± 3.8	42.2 ± 11.5	0.0032	0.0052
Anserine	Spring	8.0 ± 0.7	9.2 ± 0.7	0.0474	0.0474
	Summer	6.1 ± 1.4	11.2 ± 0.4	9.6 × 10^−5^	2.6 × 10^−4^
	Autumn	3.8 ± 0.7	13.8 ± 0.7	4.0 × 10^−7^	3.0 × 10^−7^
	Winter	3.9 ± 0.6	5.9 ± 1.2	0.0174	0.0199

## Data Availability

The original contributions presented in the study are included in the article, further inquiries can be directed to the corresponding author.

## Abbreviations

The following abbreviations are used in this manuscript:

SRM	selected reaction monitoring
LC-MS/MS	liquid chromatography-tandem mass spectrometry
PCA	principal component analysis
OPLS-DA	orthogonal partial least squares-discriminant analyses
AUC	area under the curve
ROC	receiver operating characteristic
HPLC-UV	high-performance liquid chromatography-ultraviolet
TCA	trichloroacetic acid
PEH	(R)-phenylephrine hydrochloride
IS	internal standard
FDR	false discovery rate
CI	confidence interval
